# Domain swaps of Arabidopsis secondary wall cellulose synthases to elucidate their class specificity

**DOI:** 10.1002/pld3.61

**Published:** 2018-07-10

**Authors:** Joseph Lee Hill, Ashley Nicole Hill, Alison W. Roberts, Candace H. Haigler, Ming Tien

**Affiliations:** ^1^ Department of Biochemistry and Molecular Biology The Center for Lignocellulose Structure and Formation Pennsylvania State University University Park Pennsylvania; ^2^ Department of Biological Sciences University of Rhode Island Kingston Rhode Island; ^3^ Department of Crop and Soil Sciences and Department of Plant and Microbial Biology North Carolina State University Raleigh North Carolina; ^4^ Present address: Department of Horticulture Michigan State University East Lansing Michigan 48824

**Keywords:** *Arabidopsis thaliana*, cellulose synthase, chimera, class specificity, domain swap, protein interaction, secondary cell wall

## Abstract

Cellulose microfibrils are synthesized by membrane‐embedded cellulose synthesis complexes (CSCs), currently modeled as hexamers of cellulose synthase (CESA) trimers. The three paralogous CESAs involved in secondary cell wall (SCW) cellulose biosynthesis in Arabidopsis (CESA4, CESA7, CESA8) are similar, but nonredundant, with all three isoforms required for assembly and function of the CSC. The molecular basis of protein–protein recognition among the isoforms is not well understood. To investigate the locations of the interfaces that are responsible for isoform recognition, we swapped three domains between the Arabidopsis CESAs required for SCW synthesis (CESA4, CESA7, and CESA8): N‐terminus, central domain containing the catalytic core, and C‐terminus. Chimeric genes with all pairwise permutations of the domains were tested for in vivo functionality within knockout mutant backgrounds of *cesa4*,* cesa7*, and *cesa8*. Immunoblotting with isoform‐specific antibodies confirmed the anticipated protein expression in transgenic plants. The percent recovery of stem height and crystalline cellulose content was assayed, as compared to wild type, the mutant background lines, and other controls. Retention of the native central domain was sufficient for CESA8 chimeras to function, with neither its N‐terminal nor C‐terminal domains required. The C‐terminal domain is required for class‐specific function of CESA4 and CESA7, and CESA7 also requires its own N‐terminus. Across all isoforms, the results indicate that the central domain, as well as the N‐ and C‐terminal regions, contributes to class‐specific function variously in Arabidopsis CESA4, CESA7, and CESA8.

## 1 INTRODUCTION

Cellulose is the most abundant biopolymer on earth. It plays a critical role in plant cell growth and morphogenesis, acting as one of the load‐bearing components of the cell wall and helping to regulate anisotropic growth (Cosgrove, 2014). Much remains unknown about plant cellulose, including the details of its para‐crystalline structure and biosynthesis. In contrast to the recently crystallized bacterial cellulose synthase (Morgan, Strumillo, & Zimmer, 2013), cellulose synthases from plants and some other organisms form cellulose synthesis complexes (CSCs) where multiple β‐1,4‐glucan chains are produced in close proximity as a prelude to microfibril formation (Giddings, Brower, & Staehelin, 1980; Kimura et al., 1999; Mueller & Brown, 1980). CSCs exist in a variety of structural configurations, which are thought to control cellulose microfibril structure (Itoh, Kimura, & Brown, 2007; Okuda, 2007; Tsekos, 1999). In higher plants, CSCs form hexameric, or “rosette,” structures (Mueller & Brown, 1980). Rosettes were recently proposed to comprise 18 cellulose synthase (CESA) proteins arranged in a “hexamer of trimers” configuration (Hill, Hammudi, & Tien, 2014; Newman, Hill, & Harris, 2013; Nixon et al., 2016).

In *Arabidopsis thaliana,* the CSCs that synthesize secondary cell wall (SCW) cellulose are composed of CESA4, CESA7, and CESA8 (Taylor, Howells, Huttly, Vickers, & Turner, 2003). CESAs that synthesize primary cell wall (PCW) cellulose are composed of CESA1, CESA3, and CESA6 (or 6‐like CESAs) (Desprez et al., 2007; Persson et al., 2007; Somerville, 2006). Recent studies show that in both cases, the three CESA isoforms are present in equimolar stoichiometries (Gonneau, Desprez, Guillot, Vernhettes, & Hofte, 2014; Hill et al., 2014). Characterization of cellulose‐deficient phenotypes in numerous genotypes with mutations in only one CESA shows a stringent requirement for three distinct CESAs during PCW and SCW synthesis. For example, the loss of just one SCW CESA in Arabidopsis causes complete loss of detectable SCW cellulose, with no further effect in double or triple SCW *atcesako* lines (Kumar & Turner, 2015). In addition, when one SCW *AtCESA* is knocked out, protein levels of the remaining two interacting AtCESAs are lost or severely depleted (Hill et al., 2014). The pattern of two CSCs, each with three CESAs is broadly conserved in seed plants, for which characterized genomes contain members of six phylogenetic clades that each encompass one of the required Arabidopsis CESAs (Carroll & Specht, 2011; Kumar et al., 2009).

In an effort to identify regions of the CESA proteins that might be involved in CESA–CESA interaction within the CSC, Carroll and Specht analyzed 82 CESAs from 11 plant species to identify “class‐specific regions,” that is portions of the sequence alignment that have higher similarity within versus between CESA classes (Carroll & Specht, 2011). They concluded that regions of high sequence class specificity are similar among the clades with the exception of the far N‐terminus, which is missing in the CESA3, CESA4, and CESA8 classes, and the far C‐terminus, where the CESA1 and CESA8 classes are highly divergent. To empirically test the functional significance of this sequence class specificity, Kumar and coworkers performed a number of reciprocal domain swaps with AtCESA4, AtCESA7, and AtCESA8 (Kumar, Atanassov, & Turner, 2017). Their results suggest that no one individual region is responsible for functional class specificity and that features distributed throughout CESA proteins contribute to class‐specific function.

Several regions within CESA sequences are absent from bacterial cellulose synthase and thus might contribute to the unique assembly and class specificity observed in plant CESAs. Zn‐binding RING domains are implicated in protein–protein recognition and binding (Leon & Roth, 2000). When expressed heterologously, cotton CESA Zn‐binding domains dimerize in a redox‐dependent manner (Jacob‐Wilk, Kurek, Hogan, & Delmer, 2006), suggesting one possible step of CSC assembly (Carpita, 2011). In the central domain, the Plant‐Conserved Region (P‐CR) (Pear, Kawagoe, Schreckengost, Delmer, & Stalker, 1996) is highly conserved in sequence and structure among plant CESAs (Carroll & Specht, 2011; Rushton et al., 2017; Sethaphong et al., 2016), but its functional role is not yet proven (Rushton et al., 2017; Sethaphong et al., 2016). In contrast, the Class‐Specific‐Region (CSR), as its name implies, is more similar within versus between orthologous CESA groups (Carroll & Specht, 2011; Vergara & Carpita, 2001). Similar to the N‐terminal domain, heterologously expressed truncated CESA central domains can form multimers: rice CESA8 central domains dimerized (Olek et al., 2014), whereas AtCESA1 central domains trimerized (Vandavasi et al., 2016). In addition, the crystal structure of heterologously expressed rice CESA8 P‐CR includes a 3‐fold contact. However, a trimer modeled using this contact is thought to be impossible when the membrane domain is included (Rushton et al., 2017). To date, a heterologously expressed truncated CESA C‐terminal domain has not been studied, but the chitin synthase SPSA of *Bacillus subtilis*, a homolog of CESA, requires a similar C‐terminal tail for dimerization (Charnock, Henrissat, & Davies, 2001).

**Figure 1:**
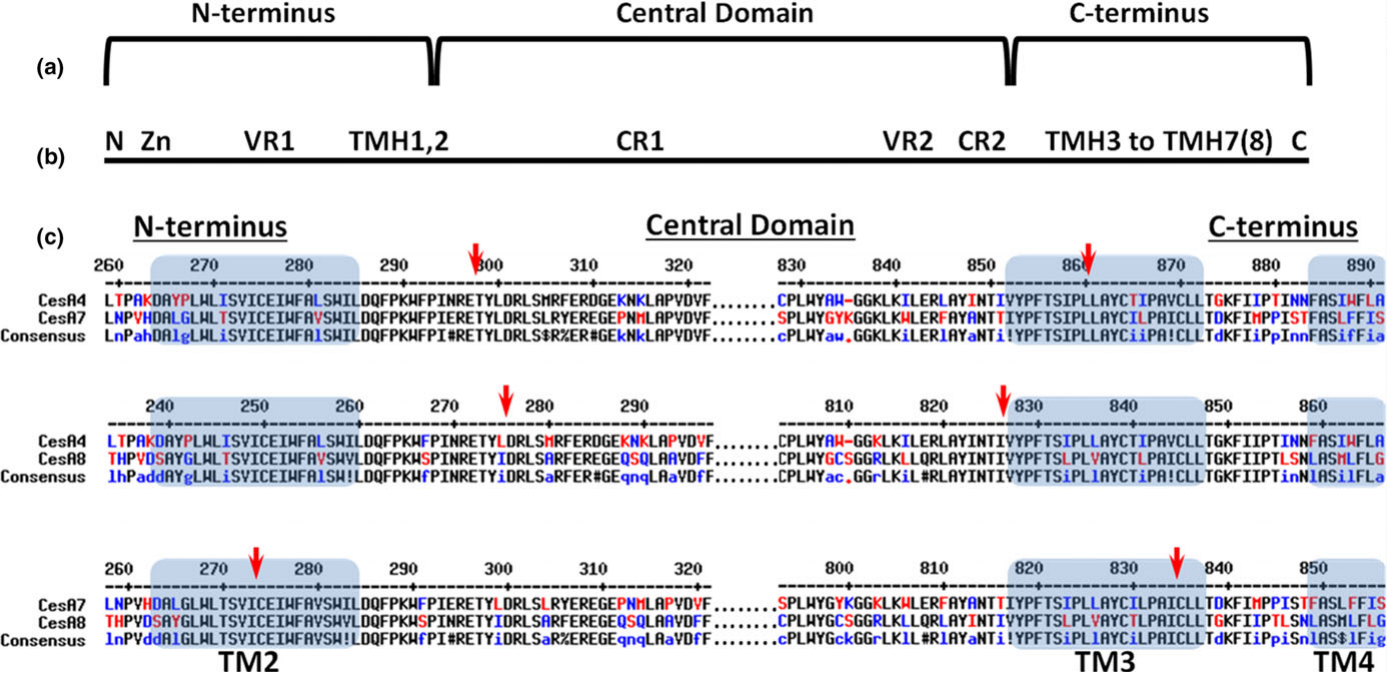
Chimeric CESA construction. (a) Three CESA segments assembled to make chimeric constructs: N‐terminus; central domain containing the catalytic core and the peripheral P‐CR and CSR domains; and the C‐terminus. (b) Nine CESA segments swapped by Kumar and coworkers (Kumar et al., 2017) to make chimeric constructs: N = short N‐terminus prior to the Zn‐binding domain; Zn = Zinc‐binding domain; VR1 = variable region 1; TMH1,2; CR1 = conserved region 1 with the P‐CR in the middle; VR2 = variable region 2 composed mostly of the CSR; CR2 = conserved region 2; TMH3‐7(8), and C = remaining protein after the last TM helix. (c) Trimmed MULTALIN alignments showing the junction (arrow) between segments assembled in chimeric constructs. These junctions were all in the vicinity of TM2 or TM3 (blue boxes), but were selected for each domain swap pair to maximize amino acid and nucleotide sequence identity adjacent to the junction

A recent study tested chimeric CESAs produced by swapping nine relatively short CESA regions (Kumar et al., 2017). In contrast, we tested fewer and generally larger protein regions (compare Figure 1a,b) for their ability to function within paralogous SCW CESAs. We reasoned that swapping larger domains could preserve functional regions within the tertiary structure of chimeric CESAs, including those that may cross the boundaries between smaller regions. As in the previous study (Kumar et al., 2017), we tested the ability of chimeric genes to rescue crystalline cellulose deficiency and short stems in the relevant knockout mutant background lines of *AtCESA4*,* AtCESA7*, and *AtCESA8 (*the *cesa4ko, cesa7ko,* and *cesa8ko* lines, respectively). In our experiments, we swapped three domains (Figure 1): (a) a N‐terminal region inclusive of a Zn‐binding RING motif (Zn), a variable sequence region (VR1), and transmembrane helices (TMH1,2); (b) a large cytosolic/catalytic central domain, which includes the P‐CR (within CR1) and CSR (within VR2); and (c) a relatively short C‐terminal region composed mostly of TMHs (Pear et al., 1996; Saxena & Brown, 1997). Our results indicate that the central domain and C‐terminus confer class specificity in AtCESAs involved in SCW cellulose synthesis. Although the N‐terminal domain appears not to be important in class‐specific interactions, this does not rule out the possibility that it participates in CESA–CESA interactions that are non‐class‐specific.

## 2 MATERIALS AND METHODS

All chemicals were obtained from Sigma‐Aldrich, St. Louis, MO unless otherwise specified.

### 2.1 Seed and DNA stocks

Seeds were obtained from the Arabidopsis Biological Research Center (ABRC, Ohio State) for wild‐type Arabidopsis of the Columbia ecotype (CS70000), *cesa4ko* (*irx5‐4*, SALK_084627), *cesa7ko* (*irx3‐4*, SALK_029940C), *cesa8ko* (*irx1‐5*, SALK_026812C) (Alonso et al., 2003). Only one of these, *cesa4ko* (*irx5*‐4) with a T‐DNA insertion in the third exon close to the N‐terminus, was used as a background mutant line by Kumar and coworkers (Kumar et al., 2017). However, all three mutant lines were described previously (Brown, Zeef, Ellis, Goodacre, & Turner, 2005) and used by Carroll and coworkers (Carroll et al., 2012) for promoter‐swap experiments and by Hill and coworkers (Hill et al., 2014) to demonstrate that they are complete null alleles. Corresponding to that, each one shows the well‐known irregular xylem (*irx*) phenotype (Turner & Somerville, 1997). CESA4 and CESA7 cDNA clones were obtained from the ABRC (stock #U50150 and #U22199, respectively). The *CESA8* cDNA clone was a gift from Ying Gu (The Pennsylvania State University). The pORE‐O3 plant transformation vector was obtained from the ABRC (stock #CD3‐922).

### 2.2 Generation of transgenic *Arabidopsis* plant lines

Promoter fragments comprising approximately 2.5 kb of sequence upstream of the CESA4 or CESA7 start sites were amplified by polymerase chain reaction with the primers listed in Supporting Information Table S1, digested with *SacII* and *NotI*, and then ligated into *SacII*/*NotI* digested pORE‐O3 (Coutu et al., 2007) to generate pORE‐O3[Pro4] and pORE‐O3[Pro7].

CESA topology was predicted with TOPCONs web server (Tsirigos, Peters, Shu, Kall, & Elofsson, 2015) to define the transmembrane (TM) regions, TM2 and TM3. Pairwise amino acid (Corpet, 1988) and cDNA (Kumar, Tamura, & Nei, 1994) alignments were made between all combinations of AtCESA4, AtCESA7 and AtCESA8 with default parameters (BLOSUM‐62 for amino acid, ClustalW with IUB matrix for cDNA). From these alignments, primers were designed to assemble chimeric CESA genes with regions of high amino acid and cDNA identity selected as the junctions between domains. CESA4 (AT5G44030; NM_123770), CESA7 (AT5G17420; NM_121748), and CESA8 (AT4G18780; NM_117994) cDNA fragments corresponding to the N‐terminus, central domain, or C‐terminus were amplified with the primers listed in Supporting Information Table S2. These purified insert pieces were then assembled via a SLiCE reaction into *NotI/PstI* digested pORE‐O3[Pro4] (for CESA4/CESA8 domain swaps) or pORE‐O3[Pro7] (for CESA4/CESA7 and CESA7/CESA8 domain swaps) (Zhang, Werling, & Edelmann, 2012).

For simplicity, the chimeric CESA genes are named according to the isomer origin of their constituent domains, for example CESA484 (or simply 484 in graphs) is composed of the N‐terminus of CESA4, the central domain of CESA8, and the C‐terminus of CESA4. The chimeric constructs were introduced by the floral dip method (Clough & Bent, 1998) into two cognate knockout lines among three possibilities: *cesa4ko* (*irx5‐4*), *cesa7ko* (*irx3‐4*), or *cesa8ko* (*irx1‐5*). For example, *CESA484* was transformed into both the *cesa4ko* and *cesa8ko* background lines to yield two novel genotypes designated as *cesa4ko* ^CESA484^ or *cesa8ko* ^CESA484^. Transgenic plants were selected by spraying soil‐grown seedlings with 2 ml of 75 μg/ml Glufosinate‐ammonium at 7, 10, and 13 days after planting. For biochemical analysis, plants at the T2 or homozygous T3 stage were typically used, and a consistent stem height phenotype was observed for at least three independent transformants of each genotype across multiple generations. The exceptions were *cesa8ko* ^CESA484^, where only a single transformant was recovered, and *cesa4ko* ^CESA484^, where a pool of two dozen T1 plants was analyzed.

To generate CESA8^ΔNT^, a fragment amplified from CESA8 cDNA with primers 5′ATCTCCGGCCGTCCCTGCGGCCGCCATGAGGACAAAAATCACTTCATATAGG3′ and 5′TCACTAGTAAAAGGTACCGAGCTCCTTAGCAATCGATCAAAAGACAGTTC3′ was inserted in pORE‐O3[Pro7] via a SLiCE reaction (Coutu et al., 2007; Zhang et al., 2012). This construct was transformed into *cesa8ko* (*irx1‐5*), and two independent transgenic lines were selected as described above.

### 2.3 Phenotype analysis

Plants were grown at 22–24°C with 18 hr days in 4″ square pots, 6 plants per pot on average, containing ProMix BX (Premier Tech Horticulture, Quakertown, PA), supplemented with Osmocote (14‐14‐14) slow release fertilizer at a rate of 3 g per liter of growing media (ScottsMiracle‐Gro, Marysvile, OH). With the exception of *cesa8ko* ^CESA484^ and *cesa8ko* ^CESA8ΔNT^, all plants were grown at the same time under identical conditions. The *cesa8ko* ^CESA8ΔNT^ and *cesa8ko* ^CESA484^ lines were grown beside wild type and *irx1‐5* for direct comparison. Normalization of cellulose content and stem height to the value of the wild type in the same experiment minimized the effects of confounding variables that could potentially have arisen from variances in different growing cycles. Stem height was measured with a ruler (minimum of 6 plants per line) as the full length of the primary inflorescence stem of 10‐week‐old plants.

Crystalline cellulose content was assessed in 10‐week‐old‐stems after dissolving other components in strong acid (Updegraff, 1969). Primary inflorescence stems of 8–20 plants per line were stripped of branches, siliques, and leaves and cut into small pieces. Tissue was extracted with 70% ethanol then 100% acetone for at least 1 day each. After removal of acetone, the tissue was air‐dried and ball milled to a fine powder at ambient temperature with a CryoMill (Retsch, Haan, Germany). Five 2–8 mg samples of each stem tissue pool were assayed independently, and standard deviations are reported for these technical replicates. Each sample was incubated in 1 ml of Updegraff reagent (8:2:1, acetic acid:H_2_O:nitric acid) for 30 min in a boiling water bath. Cooled samples were pelleted by centrifugation, washed successively with 1 ml of H_2_O and 1 ml of acetone, then air‐dried. The pellets were resuspended and completely dissolved in 1 ml of 12 M H_2_SO_4_, requiring about 16 hr. A 20‐50 μl aliquot of each sample was diluted with H_2_O to a final volume of 350 μl prior to adding 650 μl of concentrated H_2_SO_4_ containing 0.2% anthrone. The samples were boiled (5 min) alongside a glucose standard curve, then cooled. The absorbance at 620 nm of 200 μl aliquot was determined in a microplate reader. The crystalline cellulose content was calculated from the linear standard curve of glucose and expressed as a percentage of the wild‐type value. Genotypes that differed significantly in stem height and cellulose content from both the wild type and the background mutant (Mann–Whitney nonparametric test, *p* < 0.01) were interpreted as partial rescues. The Real Statistics Resource Pack (Release 5.1) for EXCEL was used for this analysis (Copyright, 2013‐2017, Charles Zaiontz, www.real-statistics.com).

### 2.4 Immunoblotting

For protein blotting, protein was extracted from 7‐week‐old primary inflorescence stems. Stems were ground in liquid nitrogen and acetone containing 10% trichloroacetic acid was added for protein extraction (Wang et al., 2006). The protein pellets were washed with acetone, dried, and then resuspended in phosphate‐buffered saline containing 1% SDS. Protein content was determined as per (Peterson, 1977). Samples for immunoblotting were diluted to 2 mg/ml in SDS‐PAGE loading buffers prior to immunoblotting as previously described (Hill et al., 2014). Antibodies were made by Covance (Denver, PA) using synthetic peptide antigens targeted to unique N‐terminal or central domain regions for each CESA, and affinity purified in our lab as previously described (Hill et al., 2014). The specificity of the antibodies was tested by western blots against heterologously expressed CESAs and extracts of wild type and knockout lines of Columbia ecotype as previously described (Hill et al., 2014). Although the individual domains were not used for western blot analysis, the antibodies were synthesized to specific domains of each CESA.

Proteins were subjected to SDS‐PAGE and then transferred to 0.1 μm pore nitrocellulose (Whatman, Piscataway, NJ). The membranes were then processed with primary antibody, and secondary antibody (Goat anti‐rabbit horseradish peroxidase conjugate, KPL 95058‐730) as described previously (Hill et al., 2014). Blots were visualized with SuperSignal West Pico Chemiluminescent Substrate and CL‐Xposure Film (both from ThermoFisher Scientific, Waltham, MA). Cropped blot images are provided in the main text figures and full‐length blots are shown in Supporting Information Figure S4.

## 3 RESULTS

### 3.1 Domain swap strategy and implementation

CESAs were divided into three large regions, the N‐terminus, central domain, and C‐terminus (Figure 1). To minimize possible complications arising from improper folding of chimeric constructs, domains were spliced at highly conserved locations in the TMH region. TMH2 was chosen as the transition between the N‐terminus and central domain and TMH3 as the transition point between the central domain and C‐terminus (Figure 1). Although it is uncertain whether particular predicted TMHs are authentic and consequently whether CESA has seven or eight TMHs (Slabaugh, Davis, Haigler, Yingling, & Zimmer, 2014), it is generally accepted that TMH2 and TMH3 form the boundaries of the large central/catalytic domain in the cytoplasm. The TMH2 transition point defines an N‐terminal region of 218–295 amino acids, including the Zn‐binding domain and a variable region. The central domain fragment corresponds to a 517–562 amino acid region containing the CESA catalytic core as well as the P‐CR and CSR regions. At last, the C‐terminal fragment consists of the remaining TMHs and the final 204–224 amino acids. Assembly of these domains in every pairwise combination between *CESA4*,* CESA7*, and *CESA8* produced 18 different constructs (Supporting Information Figure S2) named as described in the Material and Methods section. The native *CESA4* promoter was used to drive expression of the swaps between *CESA4* and *CESA8*, whereas the native *CESA7* promoter was used for the *CESA4*/*CESA7* and *CESA7*/*CESA8* swaps.

### 3.2 Rescue of *cesa8ko*

**Figure 2:**
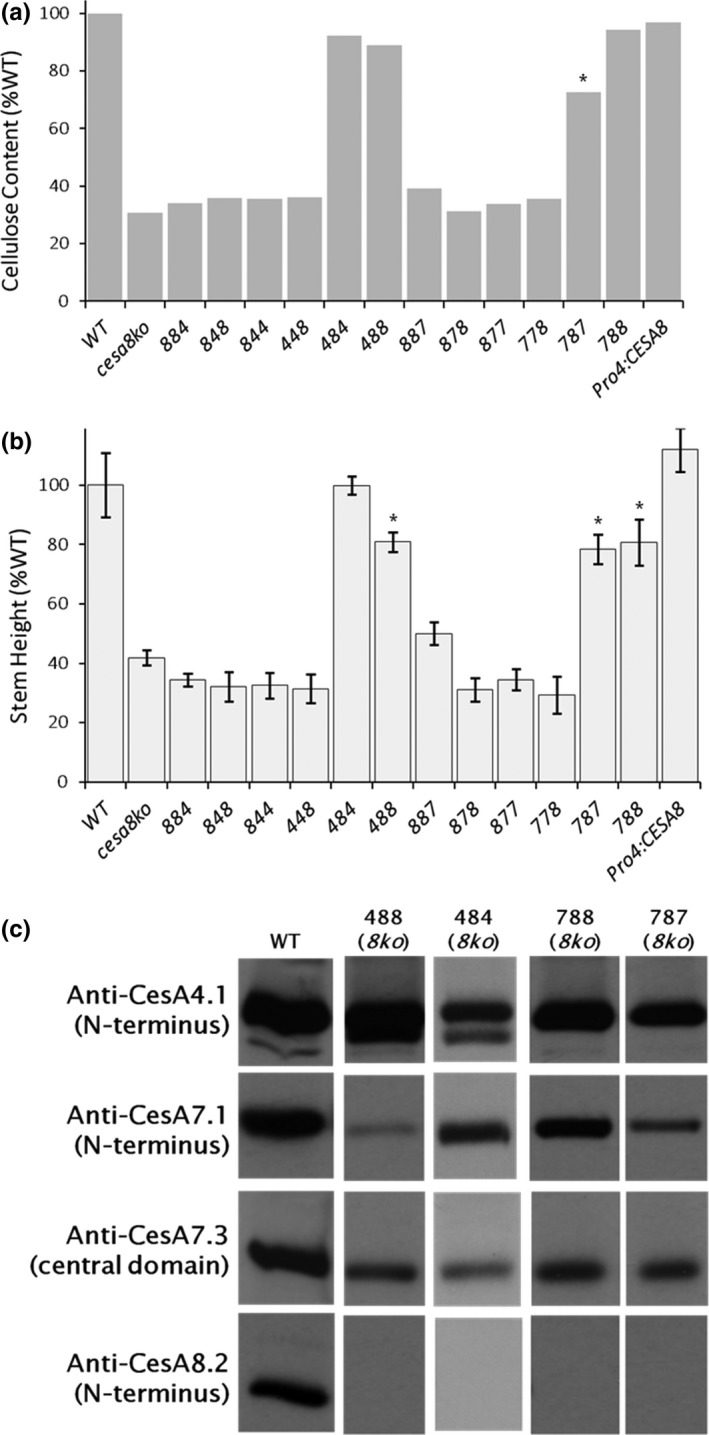
*cesa8ko* is rescued by four chimeric constructs containing the CESA8 central domain. Among 12 chimeric CESAs tested, CESA484, CESA488, CESA787, and CESA788 were able to rescue the *cesa8ko* (*irx1‐5*) phenotype. Phenotypes were also rescued by the positive control (Pro4:CESA8, *CESA8* driven by a *CESA4* native promoter). (a) Crystalline cellulose content of each genotype as a percent of wild type (WT). Error bars are standard deviations (STD) from *n* = 5 technical replicates of a pooled sample composed of primary inflorescence stems harvested from 8–20 plants per line. (b) Stem height of each genotype. STD error bars derive from *n* = 8–25 stems from individuals from a single transgenic line. In (a) and (b), asterisks indicate partially rescued lines (*p* < 0.01 as determined by nonparametric Mann–Whitney test between values for the wild type and the transformed line). (c) Immunoblot analysis characterizing domain swap lines that rescued the mutant phenotype. Anti‐CESA4.1 recognizes endogenous CESA4 in all lines and the 4 kDa smaller CESA488 and CESA484. Both anti‐CESA7.1 and anti‐CESA7.3 recognize endogenous CESA7, but CESA788 and CESA787 are too similar in molecular weight to CESA7 to be separated from the native protein. In all transgenic rescue lines, no signal from anti‐CESA8.2 was observed, confirming the *cesa8ko* genetic background

Of the 18 chimeric constructs (Supporting Information Figure S2), four at least partially restored the wild‐type phenotype in the *cesa8ko* (*irx1‐5)* background: *CESA488*,* CESA788*,* CESA484*, and *CESA787* (Figure 2 and Supporting Information Figure S2). In addition, the positive control of *CESA8* driven by the *CESA4* promoter (*Pro* ^*CESA4*^ *:CESA8*) validated the use of the *CESA4* promoter for driving *CESA8* expression. Rescue was revealed visually and quantitatively by the height of the plants (Figure 2b, Supporting Information Figure S3). The *cesa8ko* ^CESA484^ plants were the same height as wild type, whereas *cesa8ko* ^CESA488^, *cesa8ko* ^CESA787^, and *cesa8ko* ^CESA788^ plants had stem heights intermediate between wild type and the mutant background line. All of these chimeras retained the CESA8 central domain. The crystalline cellulose content in three of the transformants (*cesa8ko* ^CESA484^, *cesa8ko* ^CESA488^, *cesa8ko* ^CESA788^), was similar to wild type and the *cesa8ko* ^ProCESA4:CESA8^ control (Figure 2a). In contrast, the *cesa8ko* ^CESA787^ chimera exhibited partial rescue (73% of the wild‐type value, *p* < 0.01, Figure 2a).

To verify the genotypes of the rescued lines, we probed protein extracts (Figure 2c) with previously developed antibodies to the N‐terminus of AtCESA4 (anti‐CES4.1), AtCESA7 (anti‐CESA7.1), and AtCESA8 (anti‐CESA8.2) and central domain of AtCESA7 (anti‐CESA7.3) (Hill et al., 2014). In the *cesa8ko* ^CESA488^ and *cesa8ko* ^CESA484^ lines, anti‐CESA4.1 (N‐terminus) identifies both endogenous CESA4 and a 4 kDa smaller band corresponding to the chimeric proteins (Figure 2c). This mass‐shift is expected, as the CESA8 central domain of CESA488 and CESA484 lacks a CESA4‐specific insertion within the CSR (Supporting Information Figure S1). Endogenous CESA7 protein is recognized by both anti‐CESA7.1 and anti‐CESA7.3 (Figure 2c). Although CESA788 and CESA787 are presumably recognized by anti‐CESA7.1 (N‐terminus), they cannot be distinguished from CESA7 based on molecular mass. However, in all four lines, the absence of immunoblot signal when probing with anti‐CESA8.2 (N‐terminus) confirms the lack of a CESA possessing the CESA8 N‐terminal domain (Figure 2c).

### 3.3 Rescue of *cesa4ko*

**Figure 3:**
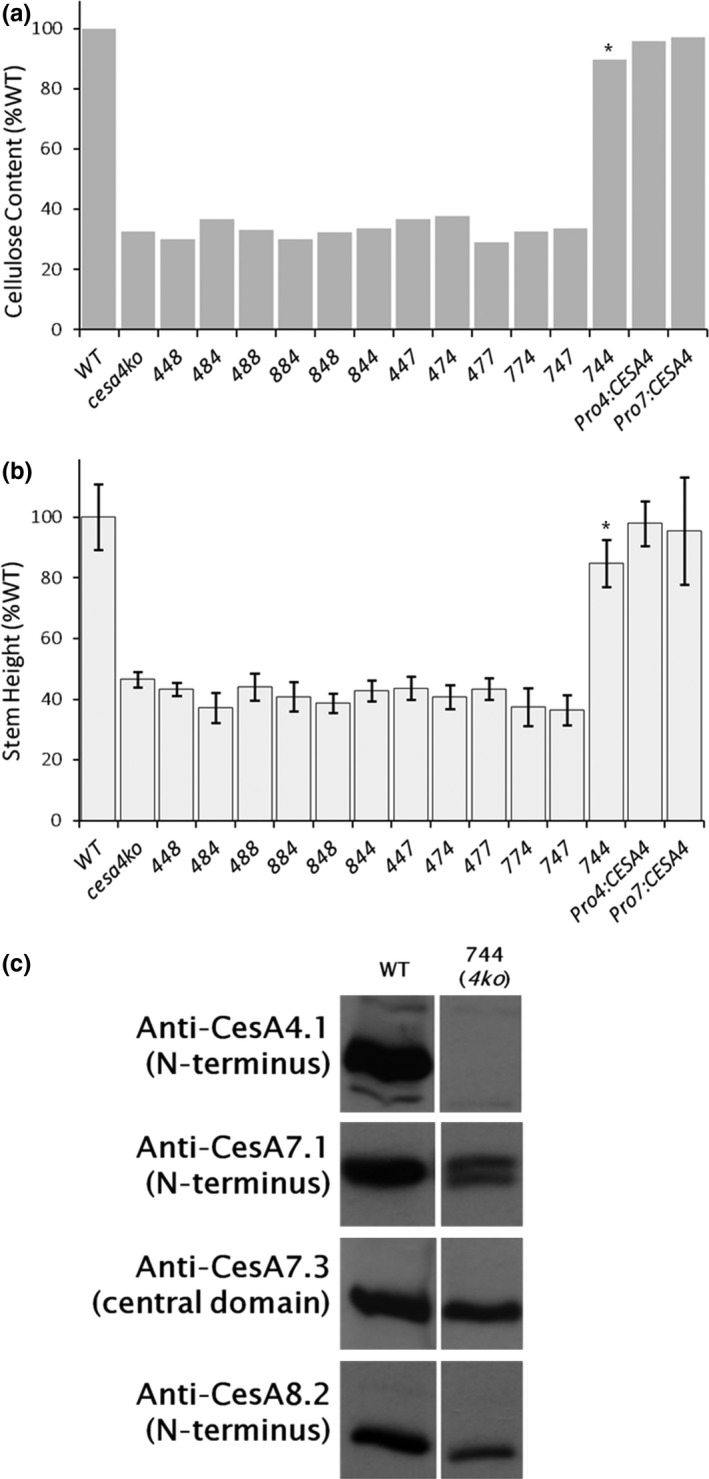
*cesa4ko* is rescued only by the CESA744 chimeric construct. Of 12 chimeric CESAs tested, only CESA744 provided phenotypic rescue of *cesa4ko* (*irx5‐4*) along with the two positive controls of CESA4 driven by native promoter fragments of CESA4 or CESA7 (Pro4:CESA4 and Pro7:CESA4). (a) Cellulose content of lines (see Figure 2 for details). (b) Stem height measurements for each line (*n* = 7–26 stems with STD error bars). In (a) and (b), asterisks indicate partially rescued lines (*p* < 0.01 as determined by nonparametric Mann–Whitney test between values for the wild type and the transformed line). (c) Immunoblot analysis showed that, as expected, no signal was observed in the rescued CESA744 line with anti‐CESA4.1, whereas a doublet was detected when probing with anti‐CESA7.1. Anti‐CESA7.3 did not recognize the 5 kDa larger CESA744, but endogenous CESA7 was recognized. Anti‐CESA8.2 confirmed the presence of CESA8

In the *cesa4ko* (*irx5‐*4) background, only the *CESA744* chimera was able to partially rescue the mutant phenotypes, restoring 85% or 89% of the wild‐type stem height or cellulose content, respectively. Both of these values are significantly different than wild type (*p* < 0.01) (Figure 3a,b). As controls, we also showed that *CESA4*, driven by *CESA4* or *CESA7* promoter fragments (Pro^CESA4^:CESA4 and Pro^CESA7^:CESA4, respectively), was able to restore both crystalline cellulose content and stem height to wild‐type levels (Figure 3a,b). When the *cesa4ko* ^CESA744^ line was characterized by immunoblot analysis (Figure 3c), CESA8 was detected with anti‐CESA8.2 and no signal arose from probing with anti‐CESA4.1 (N‐terminus), as expected in the *cesa4ko* genetic background. However, a doublet was observed with anti‐CESA7.1 (N‐terminus), where CESA744 has a 5 kDa higher mass due to the larger CESA4 CSR. In addition, the absence of a doublet when probing with anti‐CESA7.3 (central domain) signifies the absence of the CESA7 central domain in the higher molecular weight species recognized by CESA7.1 (N‐terminus), an immunoblot “fingerprint” identifying this line as *cesa4ko* ^CESA744^.

### 3.4 Rescues of *cesa7ko*

**Figure 4:**
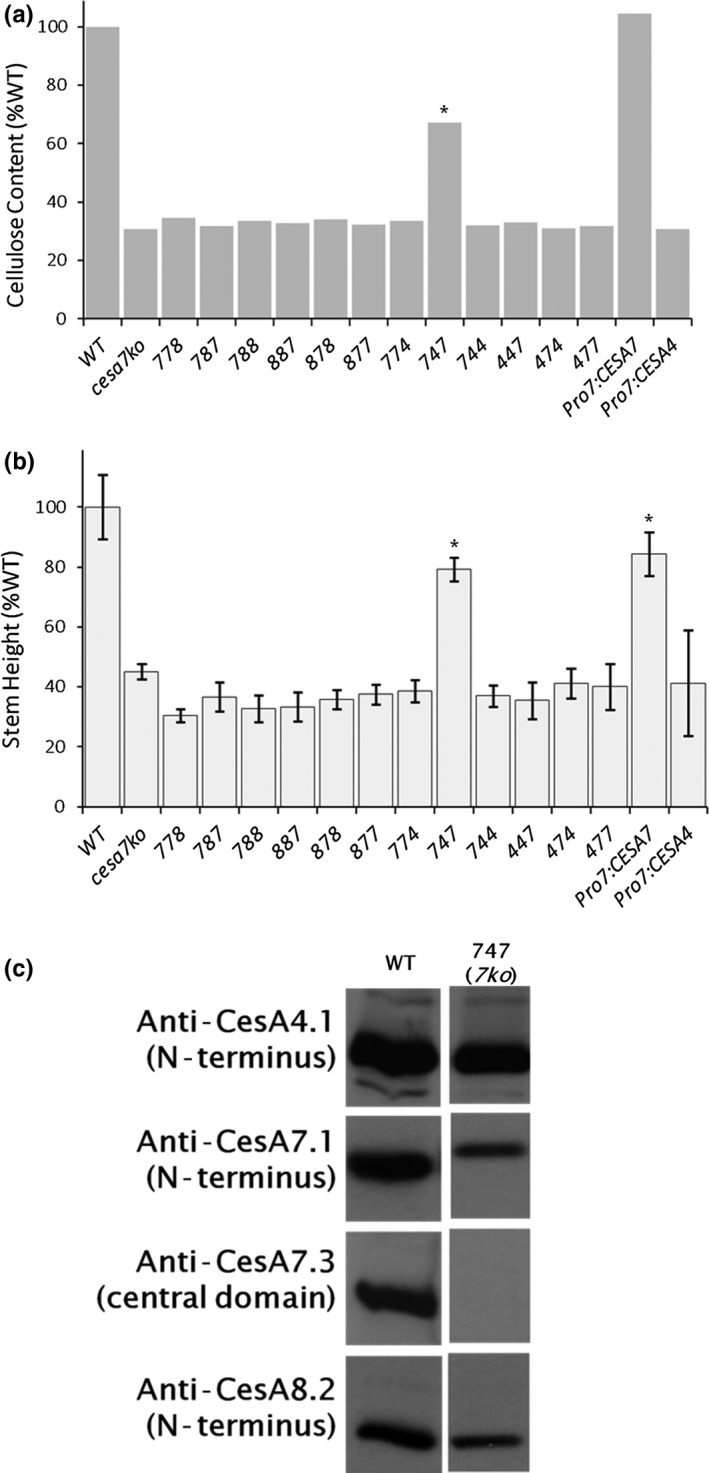
*cesa7ko* is rescued by the CESA747 chimeric construct. Of 12 chimeric CESAs, only CESA747 rescued the *cesa7ko* (*irx3‐4*) mutant phenotype, along with the positive control of Pro7:CESA7. CESA4 driven by the native CESA7 promoter (Pro4:CESA4) did not rescue. (a) Cellulose content of lines (see Figure 2 for details). (b) Stem height measurements for each line (*n* = 6–26 stems with STD error bars). In (a) and (b), asterisks indicate partially rescued lines (*p* < 0.01 as determined by nonparametric Mann–Whitney test between values for the wild type and the transformed line). (c) Immunoblot analysis showed normal CESA4 and CESA8. Furthermore, as expected, no signal was observed in the rescued CESA747 line using anti‐CESA7.3 that recognizes the central domain, while a 5 kDa larger protein is observed when probing with anti‐CESA7.1

In the *cesa7ko* (*irx3‐4*) background, only the *CESA747* chimera was able to partially rescue the mutant phenotypes, restoring 79% or 67% of the wild‐type stem height or cellulose content, respectively (*p* < 0.01) (Figure 4a,b). In the positive control of *CESA7* driven by a *CESA7* promoter (Pro^CESA7^:CESA7), crystalline cellulose content was similar to wild type although stems were shorter (*p* < 0.01). As a negative control, we expressed *CESA4* driven by a *CESA7* promoter (Pro^CESA7^:CESA4), which failed to rescue the mutant phenotypes (Figure 4a,b).

Again, immunoblotting confirmed the identity of the expressed CESA in the *cesa7ko* ^CESA747^ line (Figure 4c). Anti‐CESA4.1 and anti‐CESA8.2 confirmed the expected presence of the endogenous CESA4 and CESA8 proteins, respectively. When we probed for the CESA7.1 (N‐terminus), we observed only a single band for *cesa7ko* ^CESA747^. This cross‐reactive band is mass‐shifted upward compared to native CESA7 protein, due to the presence of the CESA4 central domain adding 5 kDa. Furthermore, probing for the CESA7 central domain (anti‐CESA7.3) gave the expected absence of signal, as CESA747 does not contain a CESA7 central domain and this is in a *cesa7ko* genetic background (Figure 2c).

### 3.5 An N‐terminal deletion of CESA8 rescues the *cesa8ko*

Our domain swaps in the *cesa8ko* (*irx1‐5*) background revealed some promiscuity in the CESA8 N‐terminal domain. The N‐terminal domain swaps using either CESA4 or CESA7 (*cesa8ko* ^CESA488^ and *cesa8ko* ^CESA788^) fully rescued crystalline cellulose content and partially rescued stem height in *cesa8ko* (Figure 2). To further determine the limits of this flexibility, we tested whether an N‐terminally truncated version of CESA8 could rescue *cesa8ko*. A methionine residue 29 amino acids before the predicted start of TMH1 was used as the translational start site for CESA8^ΔNT^, which lacks its initial 153 amino acids.

**Figure 5:**
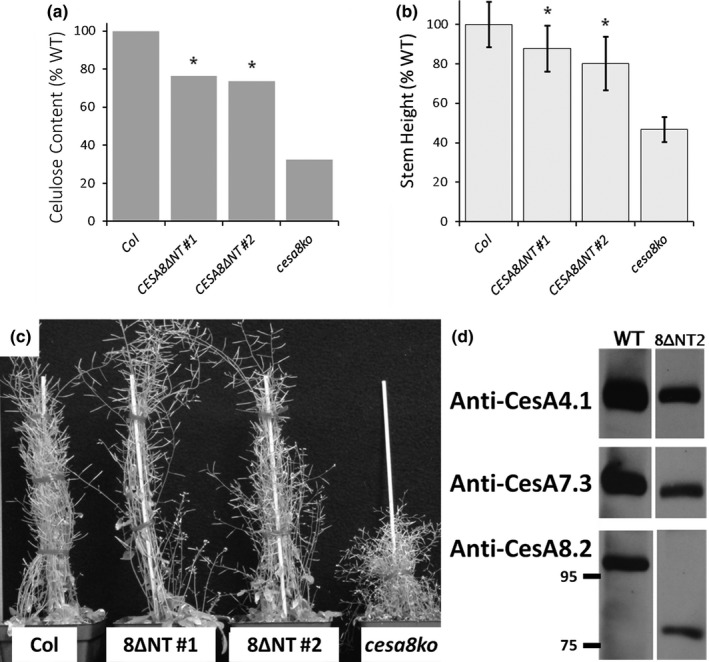
Truncation of the CESA8 N‐terminus does not abolish function. A truncated version of CESA8 (CESA8^ΔNT^) lacking 153 amino acids, or 84% of the 182 amino acids in the N‐terminus, rescued the phenotypes of *cesa8ko*. (a) Cellulose content of wild type, *cesa8ko*, and two independent CESA8^ΔNT^ transgenic lines (see Figure 2 for details). (b) Stem height measurements for each line (*n* = 24–39 stems with STD error bars). In (a) and (b), asterisks indicate partially rescued lines (*p* < 0.01 as determined by nonparametric Mann–Whitney test between values for the wild type and the transformed line). (c) Images of plants showing good phenotypic rescue with CESA8^ΔNT^ in comparison with the wild type and the cesa8ko. (d) Immunoblot analysis of CESA8^ΔNT^ showed normal CESA4 and CESA7 and that the lower MW truncated form of CESA8 can be visualized when probing with anti‐CESA8.2

CESA8^ΔNT^ provided substantial phenotypic rescue of the *cesa8ko*, as shown for two independent lines (Figure 5). Cellulose content and stem height were approximately 75% and 85% of wild‐type values, respectively (*p* < 0.01). This level of recovery is similar to that of *cesa8ko* ^CESA787^ (Figure 2). The initial methionine of CESA8^ΔNT^ lies within the middle of the epitope used to generate anti‐CESA8.2. In a fortunate way, we found that the remaining portion of the anti‐CESA8.2 epitope was sufficient to visualize CESA8^ΔNT^ during immunoblotting. Figure 5d clearly shows the absence of full‐length CESA8 and the presence of the substantially lower molecular weight CESA8^ΔNT^, as well as normal CESA4 and CESA7. Our results show that the N‐terminus of CESA8 can be removed with only moderate to low impacts on CESA8 functionality.

## 4 DISCUSSION

**Figure 6:**
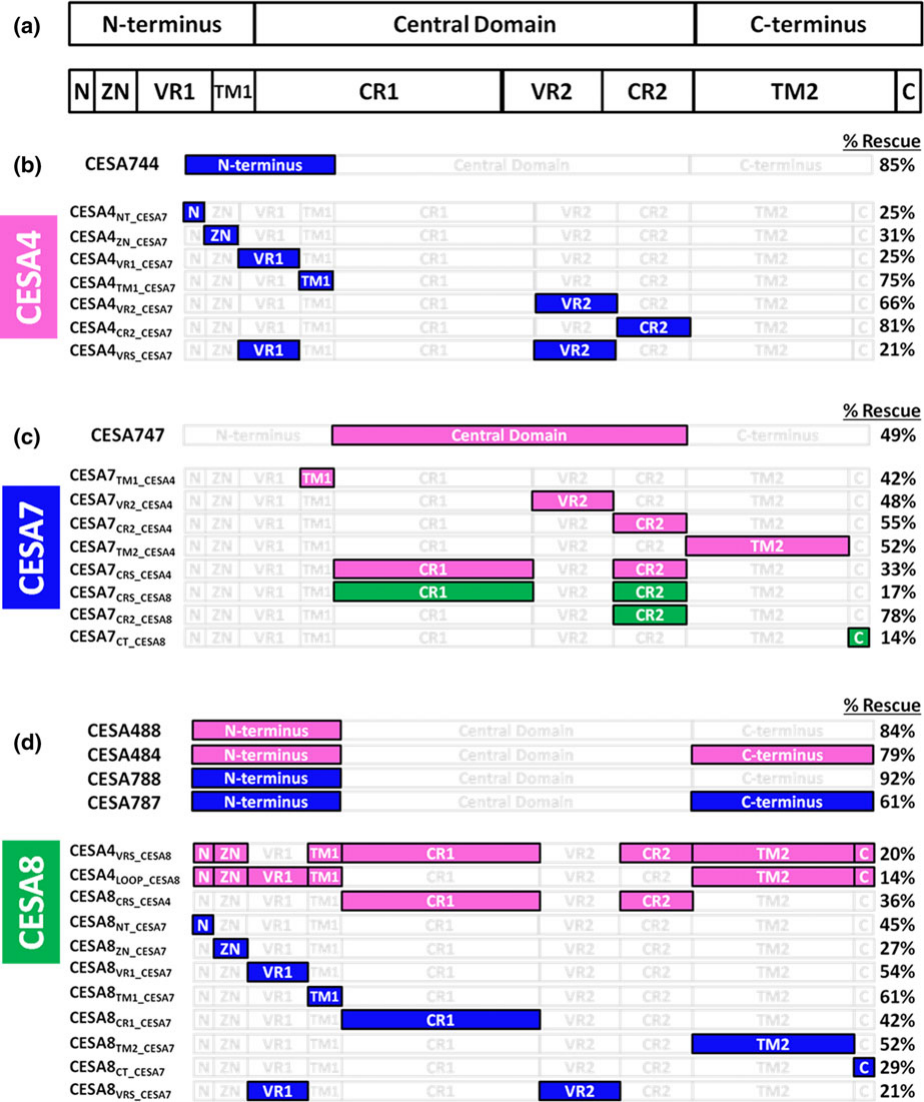
Diagram illustrating chimeric constructs complementing knockout mutants in two studies. The positive results of the current work are compared with those reported by Kumar and coworkers (Kumar et al., 2017). Panel A illustrates the three domains used in the current work and how they align with those swapped previously (Kumar et al., 2017). The % Rescue shown refers to crystalline cellulose content, using the previously published method of subtracting the residual amount in the knockout lines from the wild‐type value before normalizing the values of other lines (Kumar et al., 2017). Swapped/donor CESAs are color coded: CESA4 (magenta), CESA7 (blue), or CESA8 (green), whereas unchanged regions are white. All constructs able to rescue each knockout line are shown in Panel b, for *cesa4ko*; Panel c for *cesa7ko*; and Panel d for *cesa8ko*. Note that this figure is conceptually useful, but the % rescue values are not precisely comparable because the transgenic lines studied by Kumar and coworkers (Kumar et al., 2017) were not homozygous

Using the metrics of stem length and crystalline cellulose content, our results demonstrate a limited ability of chimeric CESA genes to rescue mutant phenotypes in knockout lines of *AtCESA4*,* AtCESA7*, and *AtCESA8*. Of 18 chimeric genes we tested with swaps between isomers of the N‐terminal, Central, and C‐terminal domains (Figure 6a, Supporting Information Figure S2), only six were able to rescue the *cesa4ko* (Figure 6b), *cesa7ko* (Figure 6c) or *cesa8ko* (Figure 6d) mutant background lines (Supporting Information Figure S3). Linear regression demonstrated a strong positive correlation (*R* = 0.92, *R* ^2^ = 0.84) between stem length and cellulose content for mutant background and rescued lines (Supporting Information Figure S5). Among the rescued genotypes, 67% of wild‐type crystalline cellulose was the lowest amount that facilitated partial rescue of stem height (79% of wild‐type for CESA747 rescuing the *cesa7ko*).

The successful complementation experiments reported in our current study and the related study by Kumar and coworkers (Kumar et al., 2017) are summarized in Figure 6. The goal of the present study was to identify portions of the CESA proteins that are important in protein–protein interactions as revealed by successful complementation. Although failure of complementation is potentially informative, improper folding of a transgenic protein is difficult to distinguish from its inability to integrate into CSCs, because in both cases the CSCs will fail to arrive at the plasma membrane and all SCW CESAs will be rapidly degraded (Atanassov, Pittmann, & Turner, 2009; Hill et al., 2014).

The results of the current study and the one of Kumar and coworkers (Kumar et al., 2017) are both consistent and complementary. The *cesa4ko* line was nearly fully rescued in our study by the CESA744 chimera with the entire CESA7 N‐terminus (Figure 3), whereas the prior results showed rescue of *cesa4ko* when four smaller N‐terminal sub‐domains (N, ZN, VR1, or TM1) were swapped (Kumar et al., 2017) (Figure 6b). Similar to that, our results showed partial rescue of *cesa7ko* with CESA747 containing the entire central domain of CESA4 (Figure 4), which is consistent with results from testing swaps of the small VR2 and CR2 domains, as well as CR1 together with CR2 (Kumar et al., 2017) (Figure 6c). Rescue of *cesa8ko* by four chimeras (488, 484, 788, and 787) in our study (Figure 2) showed that CESA8 could function with the N‐terminus from CESA4 or CESA7, and with either of these together with the C‐terminus from the same isomer. Kumar and coworkers (Kumar et al., 2017) likewise showed that diverse chimeras containing parts of either CESA4 or CESA7 could replace CESA8 (Figure 6d). This is consistent with phylogenetic evidence that AtCESA8 is more specialized than AtCESA7 (Scavuzzo‐Duggan et al., 2018). These results further suggest that AtCESA8 is more specialized than AtCESA4, as no chimeric constructs containing any portion of AtCESA8 were able to rescue *cesa4ko* or *cesa7ko*.

### 4.1 Functionality of CESA N‐terminal domains

Among the three CESA domains swapped in this study, the N‐terminus is the least similar between isoforms in both sequence identity (Supporting Information Table S3) and size. CESA7 has a long N‐terminus (Carroll & Specht, 2011), whereas the N‐termini of CESA4 and CESA8 are shorter by 23 and 54 amino acids, respectively. The longer CESA7 N‐terminus also shares the highest sequence similarity with the N‐termini of CESAs from the moss *Physcomitrella patens* (Carroll & Specht, 2011), which represents a lineage that diverged from the other land plants prior to the diversification of the CESA family (Roberts & Bushoven, 2007). Within the lineage that includes ferns and seed plants, the CESA7 clade diverged first, followed by divergence of the CESA4 and CESA8 clades (Carroll & Specht, 2011; Kumar et al., 2016; Yin, Johns, Cao, & Rupani, 2014). This is consistent with shortening of the N‐terminus within the lineage that includes CESA4 and CESA8. In an interesting manner, the short N‐terminus of CESA8 could be replaced by an N‐terminus from either CESA4 or CESA7 (Figure 2), or even partially deleted (Figure 5), without abolishing the ability to rescue *cesa8ko*. This indicates that the CESA8 N‐terminus is not critical for CSC function in vivo.

In an interesting manner, recent work on heterologously expressed poplar CESA8 demonstrated that the N‐terminus could be deleted with little to no effect on catalytic activity (Purushotham et al., 2016). However, the in vitro‐synthesized cellulose was more acid‐labile (less crystalline) and globular particles (potential CESA complexes) that were seen in in vitro controls were no longer observed. This suggests that the N‐terminus may indeed be involved in homomeric CESA8‐CESA8 interactions in vitro. However, these in vitro results cannot be directly compared to *in planta* results because CESA4 and CESA7 were absent and rosette CSCs were not observed in vitro. In a potential way, the poplar CESA8 N‐terminus facilitates homomeric CESA–CESA interactions in vitro that are not essential for the in vivo function of its Arabidopsis orthologue within a heteromeric CSC.

Reflecting differences between CESA isomers, bioinformatic analyses (Carroll & Specht, 2011) and other domain swapping experiments (Kumar et al., 2017) led to the conclusion that the N‐terminus of CESA7 contributes substantially to class specificity. Our observation that the *cesa7ko* could not be rescued by any chimeric CESA lacking a CESA7 N‐terminus (i.e., CESA477 or CESA877) supports the functional significance of this domain. However, the general ability of the N‐terminus to act as a class‐specific determinant is called into question by the results of *cesa4ko* ^CESA744^, where the CESA7 N‐terminus does not prevent CESA744 from functioning as a CESA4 protein.

Although our results show that the CESA7 N‐terminus is not itself sufficient to confer CESA7 class specificity, we hypothesize that it retains an ancestral domain or motif that is essential for function of the rosette CSC as a whole and that this domain/function has been lost, or partially lost, in CESA4 and CESA8. Phosphoproteomics has shown that CESA1 and CESA3 of the PCW CSC as well as CESA4 and CESA7 of the SCW CSC are phosphorylated in their N‐terminal domains (Chen, Ehrhardt, & Somerville, 2010; Jones et al., 2016; Sanchez‐Rodriguez et al., 2017; Taylor, 2007). The CESA5 N‐terminus is also phosphorylated (Bischoff et al., 2011; Nuhse, Stensballe, Jensen, & Peck, 2004). Multiple experiments with PCW CESAs using site‐directed mutagenesis to mimic the presence or absence of phosphorylation have shown that phosphorylation is able to regulate the activity of the CSC (Bischoff et al., 2011; Chen et al., 2010, 2016; Sanchez‐Rodriguez et al., 2017). In addition, experiments involving phosphorylation of the CESA7 N‐terminal domain indicate a possible role in protein stability (Taylor, 2007). Additional work will be required to precisely determine the function of the CESA N‐terminal domain in different isomers.

### 4.2 The central and C‐terminal domains provide CESA class specificity

Of the six chimeric CESAs that rescued a mutant phenotype, one did so with a mismatched central domain: *cesa7ko* ^CESA747^ was partially rescued (Figure 6c). Thirteen vectors failed to rescue when the central domain matched the CESA that was knocked out, including *cesa4ko* ^CESA747^. Although we cannot confidently interpret results for vectors that failed to rescue any mutant, results overall indicate that the central domain is not solely responsible for class‐specific function of SCW CESAs. Indeed, partial rescue of the *cesa7ko* only by CESA747 supports the importance of the N‐ and C‐terminal domains in the class specificity of AtCESA7 (Figure 4). Due to the nonrescue reported for a similar construct (CESA7_LOOP_CESA4_) previously (Kumar et al., 2017), the results for three independent lines are shown in Supporting Information Figure S6. The differences between the two studies could be explained by factors such as details of chimeric gene splicing locations, as CESA747 contains additional CESA4 regions compared to CESA7_LOOP_CESA4_. This finding was crucial for our conclusion that both the central and C‐terminal domains confer class specificity between SCW AtCESAs.

CESA8 is clearly differentiated from CESA4 and CESA7 by its central domain, as both CESA484 and CESA787 rescue the *cesa8ko* (Figure 6d). But, the central domain cannot be responsible for determining class specificity between CESA4 and CESA7, as CESA747 rescued the *cesa7ko*. Whereas a combination of factors in the N‐ and C‐termini could differentiate CESA4 and CESA7, we propose that the primary determinant(s) lies in the C‐terminal domain because switching the C‐terminal domain alters class specificity for chimeras of CESA4 and CESA7, with CESA747 able to replace only CESA7 (Figure 4) and CESA744 able to replace only CESA4 (Figure 3). Thus, our data support a model where the C‐terminal domain is critical in differentiating between CESA4 and CESA7. However, this role of the C‐terminus cannot be extended to CESA8, as both CESA484 and CESA787 function as CESA8 proteins.

Overall, the results indicate that the determinants of Arabidopsis CESA class specificity do not reside in a single region, consistent with conclusions of others (Carroll & Specht, 2011; Kumar et al., 2017) and illustrated for the two sets of domain swap experiments in Figure 6. Portions of CESA7 are able to replace the corresponding CESA4 region, with the exception of the CR1, TM2, and “C” domains as defined by Kumar and coworkers (Figure 6b) (Kumar et al., 2017). The central domain and TM regions of CESA7 can be replaced by those of CESA4, leaving only parts of the CESA7 N‐terminus and “C” domain as reservoirs of class specificity. Select CR regions and the “C” domain of CESA8 are able to substitute within CESA7 (Figure 6c). Extensive flexibility is observed in the CESA8 N‐terminal and C‐terminal domains, with the analogous domains of either CESA4 or CESA7 able to substitute (Figure 6d). Within the central domain, the CR1 domain of CESA8 also lacks class specificity (Kumar et al., 2017). This leaves only VR2 and CR2, which harbor regions that differentiate CESA8 from CESA4 and CESA7, respectively.

Current hypotheses of protein complex evolution predict that CESA class specificity involves multiple interfaces that arose sequentially (Doolittle, 2012; Finnigan, Hanson‐Smith, Stevens, & Thornton, 2012). According to that, the requirement for three CESA isomers to form typical PCW and SCW rosette CSCs is predicted to be the outcome of gene duplication followed by accumulation of neutral mutations that generated interfaces between paralogs and abolished interfaces between identical subunits (Doolittle, 2012; Finnigan et al., 2012). Therefore, single CESA isomers became unable to form homomeric CSCs, leading to the requirement of three nonredundant and class‐specific CESA isomers, even while each of them could independently synthesize a ß‐1,4‐glucan chain. Our positive rescue results are consistent with a major role of the central domain in defining AtCESA8 class specificity, whereas the C‐terminus differentiates AtCESA7 from AtCESA4. This variability between isomers in the regions (or residues) determining their class specificity is consistent with evolution of the hetero‐oligomeric state through accumulation of neutral mutations that generate interfaces between distinct subunits. The results of Kumar and coworkers (Kumar et al., 2017) and those currently reported are complementary and provide a cumulative foundation for future work in mapping the CESA–CESA interaction interfaces in the Arabidopsis SCW CSC.
