# Supplementary material for: Domain swaps of Arabidopsis secondary wall cellulose synthases to elucidate their class specificity
Source: Plant Direct. 2018 Jul 10;2(7):e00061. doi: 10.1002/pld3.61 (PMC6508838; doi:10.1002/pld3.61)
Supplement: Supplementary file 1 [file PLD3-2-e00061-s001.pdf]

Supporting Information

The Central and C-Terminal Domains of Arabidopsis Secondary Wall Cellulose Synthases Mediate their Class Specificity

Joseph Lee Hill Jr., Ashley Nicole Hill, Alison Roberts, Candace H. Haigler and Ming Tien

Table S1 – Primers for CESA promoter cloning

CesA4ProSacIIF	ACCGCGGACCCATACATGTGATGCTA
CesA4ProNotR	CAGCGGCCGCGGCGAGGTACACTGAGCT
CesA7ProSacIIF	ACCGCGGGACGAGGCAAGAGTATGTTG
CesA7ProNotR	ACGCGGCCGCAGGGACGGCCGGAGATTA

SacII and *NotI* sites underlined

Table S2 – Domain swap primers

**CesA4 + CesA8**	
4-NT-F(4/8)	CGAGAGCTCAGTGTACCTCGCCGCGGCCGCCATGGAACCAAACACCATGGCCAG
4-NT-R(4/8)	TTCGAATCTTGCGGATAACCGGTCAAGGTAGGTTTCTCGGTTAATCGGAAACCA
4-Central-F(4/8)	TCCTATTAACCGAGAAACTTACATCGACCGTCTCTCTATGAGATTTGAACGGGAT
4-Central-R(4/8)	AGAGGCAAAGAAGTGAATGGGTAGACAATTGTGTTGATGTAAGCAAGACGTTCTAGAAT
4-CT-F(4/8)	TAGCTTATATAAACACTATTGTTTATCCTTTCACTTCTATTCCTCTACTTGCCTATT
4-CT-R(4/8)	GGATATCACTAGTAAAAGGTACCGAGCTCCTTAACAGTCGACGCCACATTGCTTC
8-NT-F(4/8)	CGAGAGCTCAGTGTACCTCGCCGCGGCCGCCATGATGGAGTCTAGGTCTCCCATCTGCA
8-NT-R(4/8)	AATCTCATAGAGAGACGGTCGATGTAAGTTTCTCGGTTAATAGGAGACCATTTAGG
8-Central-F(4/8)	TCCGATTAACCGAGAAACCTACCTTGACCGGTTATCCGCAAGATTCGAA
8-Central-R(4/8)	AGTGAAAGGATAAACAATAGTGTTTATATAAGCTAATCTCTGGAGCAACTTGAGG
8-CT-F(4/8)	ACGTCTTGCTTACATCAACACAATTGTCTACCCATTCACTTCTTTGCCTCTTGTT
8-CT-R(4/8)	TCACTAGTAAAAGGTACCGAGCTCCTTAGCAATCGATCAAAAGACAGTTCAGAGAAAG
**CesA4 + CesA7**	
7-NT-F(4/7)	CGAGAGCTCAGTGTACCTCGCCGCGGCCGCCATGGAAGCTAGCGCCGGTCTTG
7-NT-R(4/7)	TAGAGAGACGGTCAAGGTAGGTCTCACGTTCAATAGGGAACC
7-Central-F(4/7)	GGTTTCCGATTAACCGAGAAACCTATCTAGATCGGCTTTCCC
7-Central-R(4/7)	CCGGAATAGTACAATAGGCAAGAAGTGGTATAGATGTGAAGGGG
7-CT-F(4/7)	GTTTATCCTTTCACTTCTATTCCTCTACTTGCCTACTGTATCCTTCCAG

7-CT-R(4/7)	GGATATCACTAGTAAAAGGTACCGAGCTCCTCAGCAGTTGATGCCACACTTG
4-NT-F(4/7)	CGAGAGCTCAGTGTACCTCGCCGCGGCCGCCATGGAACCAAACACCATGGCCAG
4-NT-R(4/7)	GGGAAAGCCGATCTAGATAGGTTTCTCGGTTAATCGGAAACC
4-Central-F(4/7)	GGTTCCCTATTGAACGTGAGACCTACCTTGACCGTCTCTCTA
4-Central-R(4/7)	CTGGAAGGATACAGTAGGCAAGTAGAGGAATAGAAGTGAAAGGATAAAC
4-CT-F(4/7)	CCCCTTCACATCTATACCACTTCTTGCCTATTGTACTATTCCGG
4-CT-R(4/7)	GGATATCACTAGTAAAAGGTACCGAGCTCCTTAACAGTCGACGCCACATTGCTTC
**CesA7 + CesA8**	
7-NT-F(7/8)	CGAGAGCTCAGTGTACCTCGCCGCGGCCGCCATGGAAGCTAGCGCCGGTCTTG
7-NT-R(7/8)	CAGCAAACCAAATCTCACAGATCACAGAGGTCAGCCATAAT
7-Central-F(7/8)	GGTTTATGGCTAACATCTGTGATATGTGAAATCTGGTTCGCTG
7-Central-R(7/8)	AATTTGCCGGTAAGAAGGCAGATGGCTGGAAGGATACAGTAG
7-CT-F(7/8)	GCTTACTGTACTCTTCCAGCTATTTGTCTCCTTACTGACAAATTCATC
7-CT-R(7/8)	GGATATCACTAGTAAAAGGTACCGAGCTCCTCAGCAGTTGATGCCACACTTG
8-NT-F(7/8)	CGAGAGCTCAGTGTACCTCGCCGCGGCCGCCATGATGGAGTCTAGGTCTCCCATC
8-NT-R(7/8)	CAGCGAACCAGATTTCACATATCACAGATGTTAGCCATAAACC
8-Central-F(7/8)	ATTATGGCTGACCTCTGTGATCTGTGAGATTTGGTTTGCTG
8-Central-R(7/8)	GATGAATTTGTCAGTAAGGAGACAAATAGCTGGAAGAGTACAGTAAGC
8-CT-F(7/8)	CTACTGTATCCTTCCAGCCATCTGCCTTCTTACCGGCAAATT
8-CT-R(7/8)	TCACTAGTAAAAGGTACCGAGCTCCTTAGCAATCGATCAAAAGACAGTTC

Underlined DNA bases correspond to primer regions that anneal to template DNA, others bases correspond to homology regions for SLiCE cloning

Table S3 – Pair-wise comparison of domain similarity

	N-terminus		Central Domain		C-Terminus	
	I.D.	Sim.	I.D.	Sim.	I.D.	Sim.
CESA4 vs CESA7	44.2%	58.0%	70.5%	80.6%	76.3%	89.3%
CESA4 vs CESA8	36.9%	50.7%	71.6%	82.1%	76.5%	88.5%
CESA7 vs CESA8	29.8%	39.6%	74.3%	85.4%	74.0%	88.2%

Pair-wise alignment by BLAST to determine sequence identity/similarity

Supporting Figures and Legends

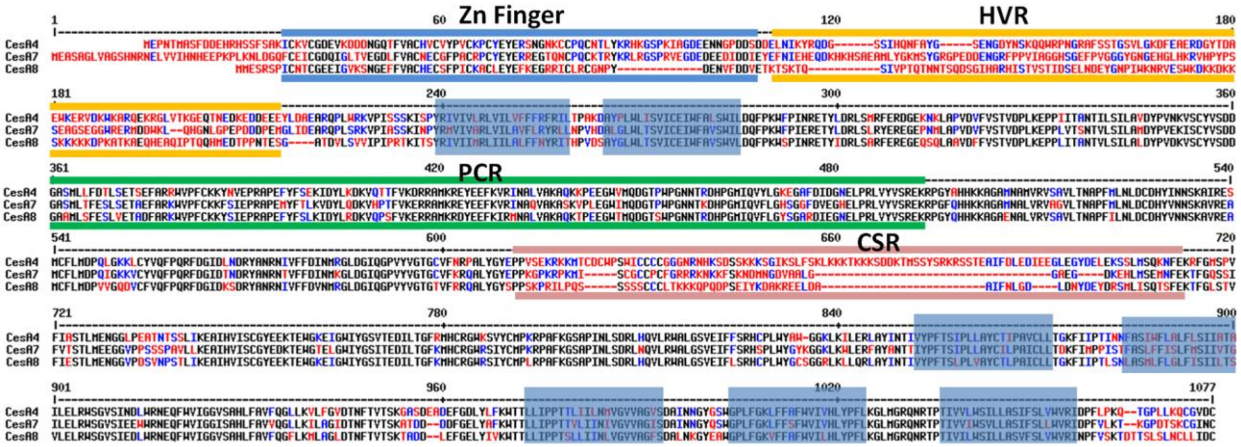

Figure S1 – Multiple sequence alignment of SCW AtCESAs.

Residues with low or no sequence conservation are marked in red, while highly conserved residues are black. CESA4, CESA7, and CESA8 share an average sequence identity of 67%, but with poorly conserved areas mainly restricted to the N-terminus and CSR regions.
Labels: Zn-finger, Hyper-Variable Region (HVR), predicted TMHs (TOPCONS prediction, shaded), Plant Conserved Region (PCR), and Class-Specific Region (CSR).

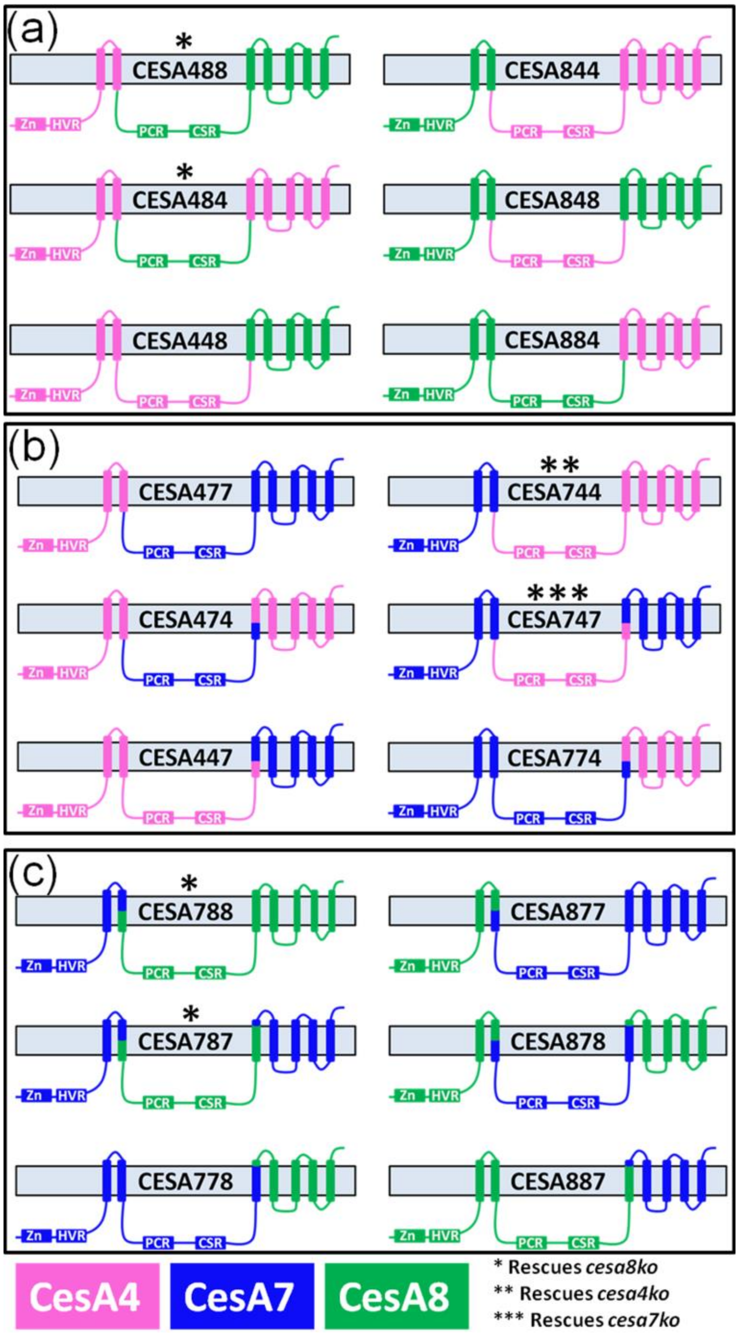

Figure S2 – CESA domain swap schematics

Schematic representation of all domain swaps that were constructed for this study with regions color coded for origin of sequence: magenta (CESA4), blue (CESA7), or green (CESA8). a) Swaps between CESA4 and CESA8, b) swaps between CESA4 and CESA7, and c) swaps between CESA7 and CESA8. Constructs rescuing *cesa8ko* marked with an asterisk (*), *cesa4ko* with a double asterisk (**) and *cesa7ko* with a triple asterisk (***).

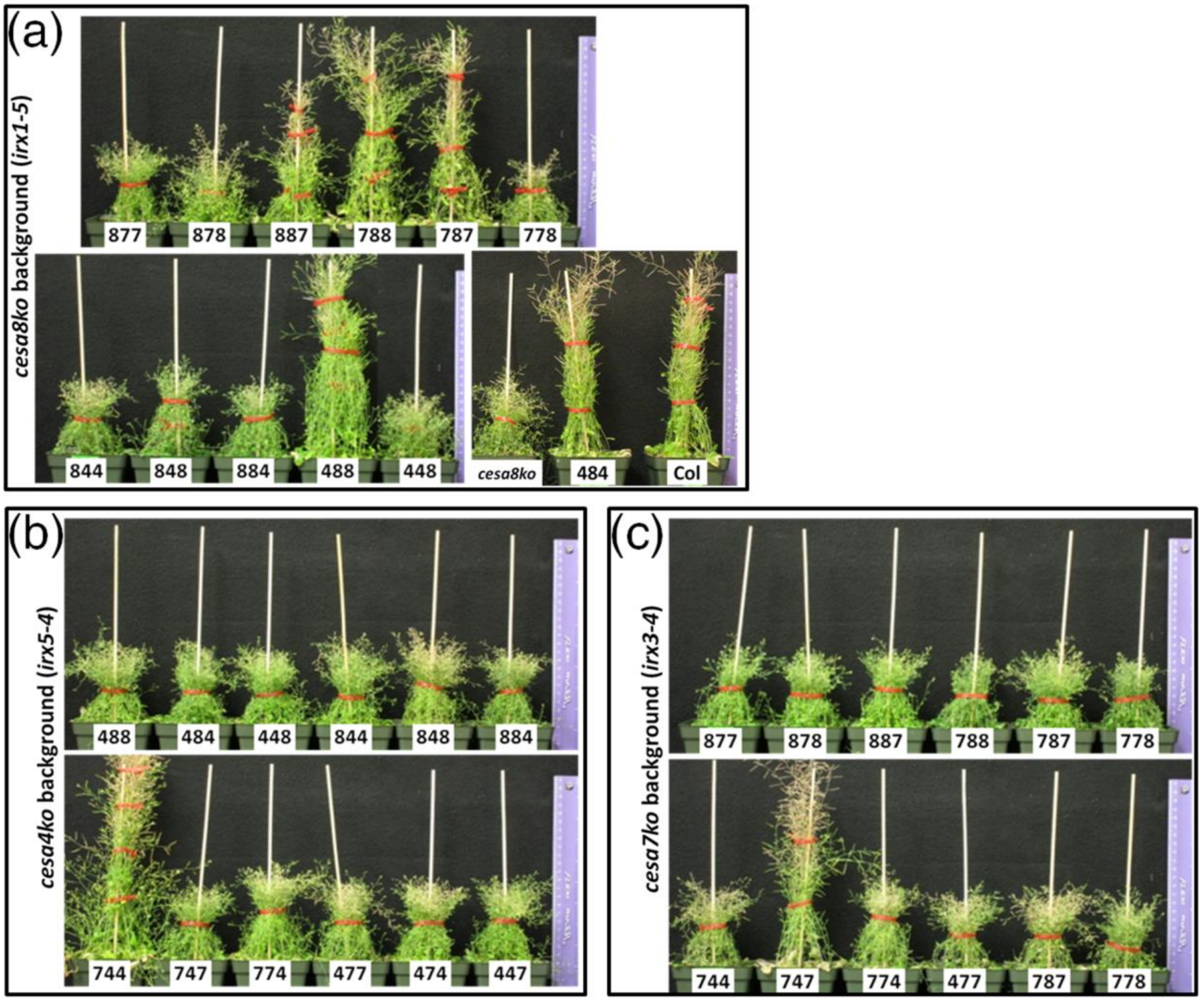

Figure S3 – Representative image of domain swap Arabidopsis lines

All plants were grown at the same time under the same conditions, with the exception of CESA484^{cesa8ko,} which was grown at a later date (pictured with wild type, for comparison). (a) Lines in the *cesa8ko* background, (b) *cesa4ko* background, and (c) *cesa7ko* background.

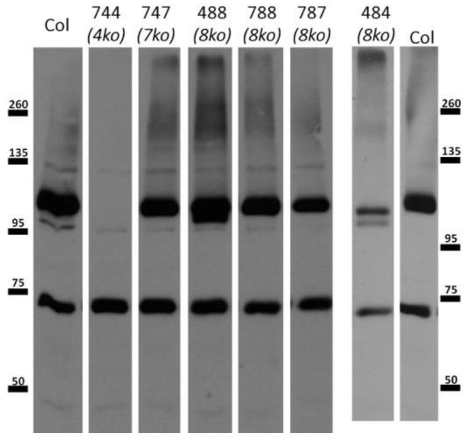

a. Anti-CesA4.1 (N-terminus)

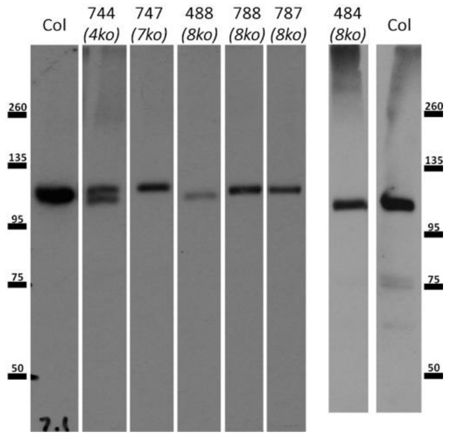

b. Anti-CesA7.1 (N-terminus)

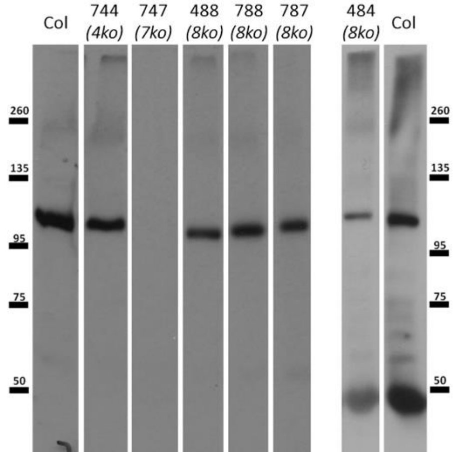

c. Anti-CesA7.3 (Central Domain)

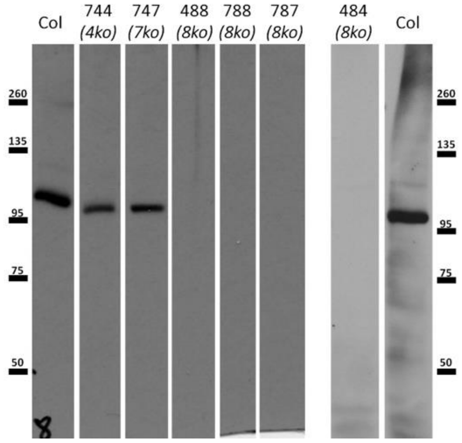

d. Anti-CesA8.2 (N-terminus)

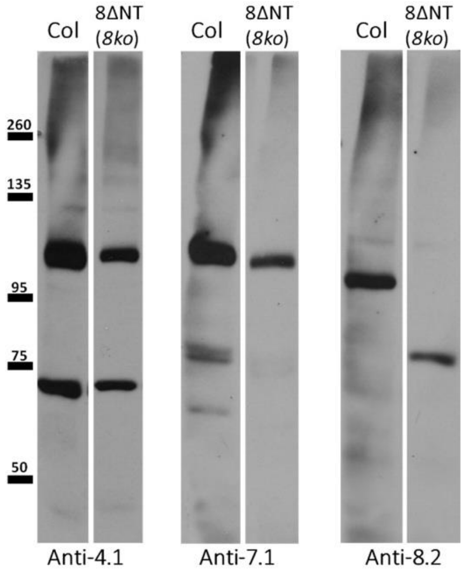

e. Analysis of CESA8ΔNT

Figure S4. Full-length immunoblots allow better assessment of antibody specificity and molecular weights of the recognized CESA proteins. These images were cropped and rearranged for Figures 2, 3, 4, and 5 in the main text. Here, chimera samples are sorted by the probing antibody: a) Anti-CesA4.1; b) Anti-CesA7.1; c) Anti-CesA7.3; and d) CesA8.2. e) Full length blots for samples analyzing the N-terminal truncated CESA8. Labels match those in Figures 2-5 and can be used to match up lanes. For example, the first two lanes in a, b, c, and d were compiled to generate main text Figure 3c.

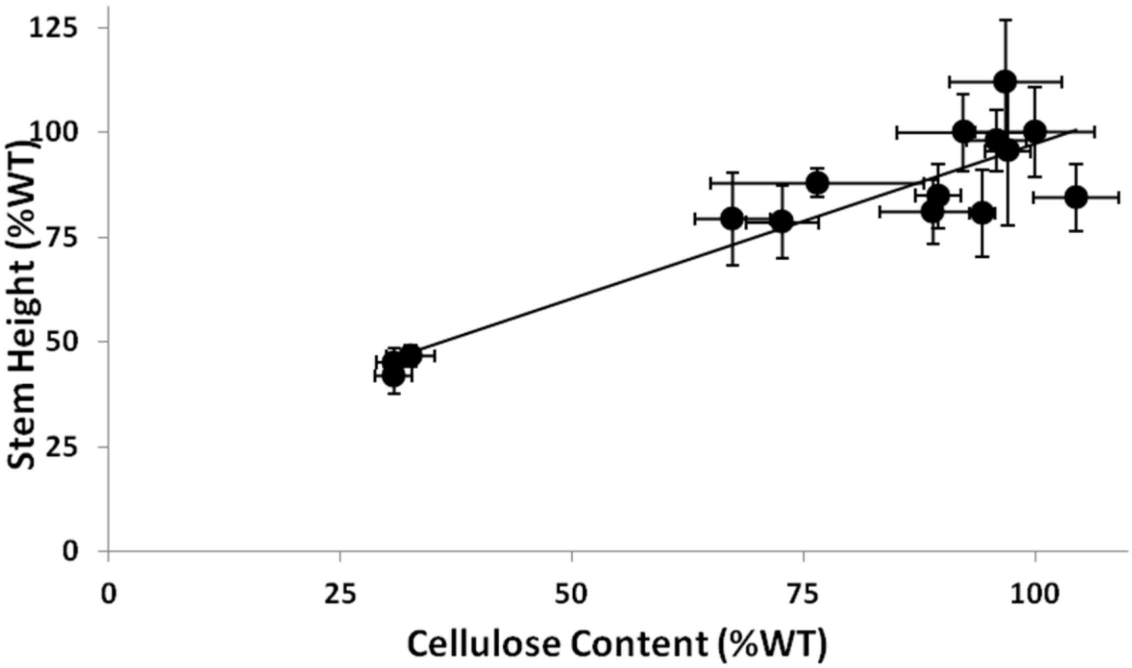

Figure S5 – Correlation of cellulose content and stem height in rescued lines.

Plot of cellulose content versus stem height of wild type, *cesako* lines, and each transgenic line displaying rescue of these phenotypes. Linear regression fit displays a strong correlation between these two phenotypes, with R = 0.92 and R^2 = 0.84 R^2. Error bars represent standard deviation.

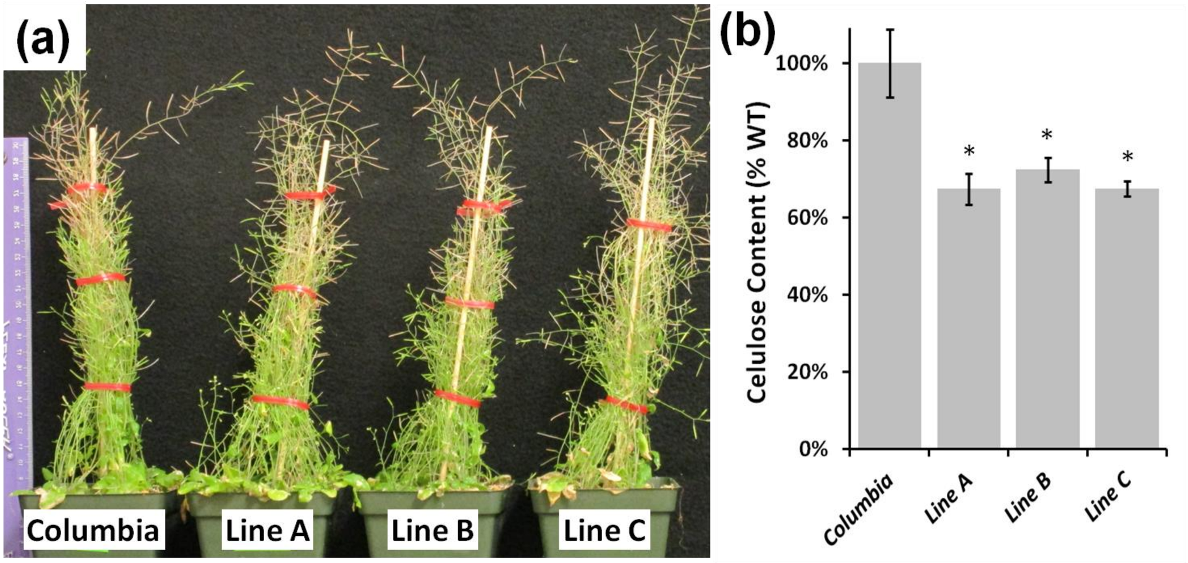

Figure S6 - *CESA747* consistently rescues the *cesa7ko*

Three independent transgenic lines of CESA747 in the cesa7ko background show consistent rescue of height (a) and cellulose content (b). Error bars represent standard deviation of five measurements. Asterisks indicate partially rescued lines ($p<0.01$ as determined by non-parametric Mann-Whitney test in comparison to wild type).
