# Supplementary material for: Domain swaps of Arabidopsis secondary wall cellulose synthases to elucidate their class specificity
Source: Plant Direct. 2018 Jul 10;2(7):e00061. doi: 10.1002/pld3.61 (PMC6508838; doi:10.1002/pld3.61)
Supplement: Supplementary file 2 [file PLD3-2-e00061-s002.pdf]

RE: Domain Swaps of Arabidopsis Secondary Wall Cellulose Synthases to Elucidate their Class Specificity

Dear Dr. Tien:

Thank you for submitting to Plant Direct. All required reviews have been returned and we have now finished our evaluation of your manuscript. In light of the reviewers' and editor's comments, further revisions are needed before the paper can be accepted for publication in Plant Direct.

Please view the editors' and reviewers' comments below and use their suggestions as a guide while you work on your revision.

When uploading the revised version of this article, please be sure to include the following:

-A word document that contains your response to the reviewers. You should respond to each reviewer comment and note the changes made to the manuscript. If you do not agree with a reviewer's comment and choose not to make a suggested revision, please explain why. Please try to provide as complete an answer as possible to each reviewer's criticisms.
-A tracked changes document with each change highlighted
- A clean version of the latest version of the manuscript

After careful review, we feel that the revised manuscript integrates many of the reviewers' previous comments, however some points to address still remain. We invite you to submit a revised manuscript that addresses the points outlined by the reviewers and academic editor.

Author's Response:
We thank the editor and the reviewers for their thorough attention to this manuscript. We appreciate the opportunity to submit a second revision, which has been reviewed and approved by all the authors. This updated version of the second revision refers to Figure 6 more extensively in order to improve the clarity of the manuscript, has been improved in general editorial ways, and, at the end of the discussion, addresses an additional request of reviewer #1 that we believe is of general interest and importance to the field (see details below). We also appreciate the currently favorable view of Reviewer #2 on the first revision and are happy to make additional changes to improve the manuscript.

1. Address Reviewer #1's comments regarding the Purushotham et al. (2016) PNAS publication (https://doi.org/10.1073/pnas.1606210113).
Author's Response: *To the existing section of the discussion, "Functionality of CESA N-terminal domains" we added information from Purshotham et al 2016 pertinent to the N-terminus. We point out differences in the in vitro and in vivo circumstances.*

2. Reviewer #2 previously noted that information in the introduction was more appropriate for the Results. The revised manuscript mostly addressed these comments. However, the text in lines 92-99 states the conceptual approach in this manuscript and is then followed by more introduction material. This text might be rephrased and relocated to the end of the Introduction.
Author's Response: *We relocated the information as requested and adjusted the phrasing to more closely match the figures in the manuscript.*

3. Supplemental Figure S4 shows the full-length western blots. Please provide a legend to this supplemental figure that provides information for each panel that links the truncated western images in the core figures to the full-length images in the supplemental figure. It would be helpful to use the same antibody naming system as is found in each of the core figures. For example, in Figure S4 panel A, "Anti 4.1" could be changed to "Anti-CesA4.1 (N-terminus)".
Author's Response: *These changes have been made.*

4. The inclusion of Figure 6 provides a comparison of results in this manuscript to those reported in Kumar et al. 2017. However, the description of this figure within a new Conclusions section seems out of

place and does not present a conclusion per se. Treatment of this figure would be better integrated within the Discussion in the appropriate place or places.

Author's response: *We appreciate the reviewer's interest in more discussion on these comparisons and have made corresponding adjustments that improve the manuscript. Figure 6 is now used as a frame of reference early in the discussion, with the focus on results that were similar to ours. We also refer to Figure 6 when appropriate later in the discussion. In the legend of Figure 6, we added a point of clarification to avoid future reader's wondering why the % cellulose values there do not exactly match our graphs. (The two studies used different methods of normalizing cellulose content, and for Figure 6 we recalculated our data according to the Kumar et al equation). No 'Conclusions' section remains in the revised manuscript.*

Other from Reviewer #1:
We noted reviewer number one's last comment that: ..." it should be discussed in the discussion how important class-specific interactions and CESA-CESA interaction for actual cellulose biosynthesis".

Author's response: *We think this is an important question at the forefront of this field, so we added three sentences to the last paragraph of the discussion to address the reviewer's request.*

Minor points to address:
Author's response:
All of these minor corrections have been made. We also corrected other minor errors or made minor stylistic improvements during our continuing work on the text. In the list below, we explain our response to a few of these items.

Line 35: Change "protein/protein" to "protein-protein"
Line 64: remove an "in" in "in in a 'hexamer of trimers configuration'
Line 81: Change "CESA/CESA" to "CESA-CESA"
Lines 133 and 134: Arabidopsis does not need to be italicized.
Line 165: The cesa4koCESA484 notation should be defined in its first use, such as "CESA484 in the cesa8ko background (i.e. cesa4koCESA484)".

Authors: *We inferred that this was a generalized request to clarify the nomenclature, but did not feel that it was necessary or wise to do it in every instance. Therefore, in Materials and Methods we provided general clarification of the nomenclature, including specific examples.*

Line 216: "peptides" should be "peptide"
Line 219: CesAs should be CESAs.
Line 219-220: "Columbia Arabidopsis" could be changed to "Columbia ecotype"
Line 226: Is the (Hill et al.) reference, Hill et al., 2014?

Lines 344-345: Plant height information is not provided in Figure S5, nor are the statistics reported.

Authors: *We mistakenly omitted the upload of actual Figure S5, which shows the correlation between the rescue of stem height and cellulose content. We also added statistical analysis to Figure S6, which shows consistency of rescue for CESA747 in the cesa7ko background.*

Lines 363-364: It is not clear how Table S3 supports this statement regarding the shorter N-terminus in CESA8.

Authors: *Table S3 was mistakenly referred to here. We now refer to Table S3 in the context of comparing sequence similarity of different domains with the secondary wall CESAs*.

Figure 2-6: The legend should clarify that the statistical test is performed between the chimera in question and wildtype.
Figure 4: Should the 747 lane in Panel C be labeled as 7 ko (not 4 ko)?
Figure 5: The lanes in panel D are not labeled as they are in previous figures [i.e. WT and NT (8 ko)].
Table S3: "Identify" should be "Identity"

Reviewer #1 :
Although I still believe that the conclusion drawn in this paper is limited due to inability to confirm that all

constructs tested were able to produce corresponding proteins, I accept the authors a statement in the text. One additional point, the authors stated in introduction that "to date, a hetorologously-expressed CESA C-terminal domain has not been studies, but ...." . However they missed the paper of Purushotham et al., 2016 in PNAS, where the authors described hetorologouse expression of populous Ces8 and demonstrated its activity and even ability to form cellulose microfiblrils, formation of which was abolished after truncation of N-terminal RING-finger domain. So conclusion in this paper should be consolidated with the results of Purushotham et al. Although the authors stated later in the introduction that "Although the N-terminal domain appears not to be important in class-specific interactions, this does not rule out the possibility that it participates in CESA-CESA interaction..." it should be discussed in the discussion how important class-specific interactions and CESA-CESA interaction for actual cellulose biosynthesis.

Reviewer #2 :
The authors have corrected my concerns. Nice study.
